# Supplementary material for: Clinical experience of genome-wide non-invasive prenatal testing as a first-tier screening test in a cohort of 59,771 pregnancies
Source: PLoS One. 2025 Aug 18;20(8):e0329463. doi: 10.1371/journal.pone.0329463 (PMC12360580; doi:10.1371/journal.pone.0329463)
Supplement: Attachment — Submitted filename: Point-by-point response.docx [file pone.0329463.s006.docx]

Dear Editor,

Manuscript Title: Clinical experience of genome-wide non-invasive prenatal testing as a first-tier screening test in a cohort of 59,771 pregnancies

Manuscript ID: PONE-D-25-18695 -[EMID:78f2caf49efcc974]

Thank you for your email regarding our submission. We are grateful for the kind feedback provided. In response to the reviewers’ comments, we have carefully revised the manuscript and addressed all the points raised. Below, we provide a point-by-point response to the reviewers’ comments and we hope that these replies and revisions meet the journal’s standards and address the concerns raised.

**Comment 1:** Please complete all items on the Human Participants Research Checklist that are relevant for your submission, by following this link: http://journals.plos.org/plosone/s/file?id=dc11/PLOSOne_Human_Subjects_Research_Checklist.docx (Contact us at plosone@plos.org if you cannot access the document.) There may be overlap between the checklist items and other queries listed below; please address any duplicated queries both in your response email and on the checklist itself. Upload the completed Human Participants Research Checklist as file type “Other” when you re-submit your manuscript.

**Reply:** We have completed all items on the Human Participants Research Checklist that are relevant for our submission and uploaded the completed Human Participants Research Checklist as file type “Other”. Meanwhile, all the approval documents obtained from the ethics committee were uploaded, covering the entire duration of the study period. The original documents are in Chinese, and the English translations have been provided for reference. We have also added an Ethics statement within the Subjects and Methods section of the manuscript, which includes the most recent ethics approval statement (LLSC-2021-04-01-10-KS) in the opening sentence to ensure compliance with ethical guidelines.

**Comment 2:** Please provide additional details regarding participant consent. In the ethics statement in the Methods and online submission information, please ensure that you have specified (1) whether consent was informed and (2) what type you obtained (for instance, written or verbal, and if verbal, how it was documented and witnessed). If your study included minors, state whether you obtained consent from parents or guardians. If the need for consent was waived by the ethics committee, please include this information.

If you are reporting a retrospective study of medical records or archived samples, please ensure that you have discussed whether all data were fully anonymized before you accessed them and/or whether the IRB or ethics committee waived the requirement for informed consent. If patients provided informed written consent to have data from their medical records used in research, please include this information

**Reply:** Thanks for your kind reminder. In this study, we obtained written informed consent from all participants, including a small number of minors, as well as from their legal guardians, prior to study enrollment. Besides, we strictly adhere to privacy protection regulations and have anonymized the data after collection on 20^th^ March 2022.

Therefore, sentence “Written informed consent was obtained from individual participants or their guardians. Records were accessed on 20^th^ March 2022 and subsequently anonymized to protect the personal privacy of all pregnant women” was added to the Ethics statement in the Subjects and Methods section of the manuscript. The content of the Ethics statement in the manuscript was added to the “Ethics Statement” field of the submission form.

The above outlines our responses to the reviewers' comments and the revisions made to the manuscript. If there are any further issues or additional revisions required, please do not hesitate to let us know.

Yours sincerely,

Jianxin Zhen

Central Laboratory

Shenzhen Baoan Women's and Children's Hospital

Shenzhen, Guangdong 518102

China

Tel: +86-075527863999

E-mail: jxzhen@qq.com
