# Supplementary material for: Clinical experience of genome-wide non-invasive prenatal testing as a first-tier screening test in a cohort of 59,771 pregnancies
Source: PLoS One. 2025 Aug 18;20(8):e0329463. doi: 10.1371/journal.pone.0329463 (PMC12360580; doi:10.1371/journal.pone.0329463)
Supplement: Attachment — Submitted filename: Response to Reviewers.docx [file pone.0329463.s007.docx]

Dear Editor,

Manuscript Title: Clinical experience of genome-wide non-invasive prenatal testing as a first-tier screening test in a cohort of 59,771 pregnancies

Manuscript ID: PONE-D-25-18695 -[EMID:66cdbb8b69d2ebbf]

Thank you for your email regarding our submission. We sincerely appreciate the detailed suggestions provided by your esteemed journal. In response to the journal requirements and reviewers’ comments, we have carefully revised the manuscript and addressed all the points raised. Below, we provide a response to reviewers and we hope that these replies and revisions meet the journal’s standards and address the concerns raised.

**Journal Requirements:**

**Requirement 1:** Please ensure that your manuscript meets PLOS ONE's style requirements, including those for file naming.

**Reply:** We have carefully revised the manuscript and the file naming according to your journal's formatting guidelines to ensure full compliance with PLOS ONE's style requirements.

**Requirement 2:** This research was supported by 2024 High-quality Development Research Project of Shenzhen Bao'an Public Hospital (Grant number: BAGZL2024154), and National Natural Science Foundation of China (Grant number: 82203291).

Please state what role the funders took in the study. If the funders had no role, please state: "The funders had no role in study design, data collection and analysis, decision to publish, or preparation of the manuscript."

If this statement is not correct you must amend it as needed.

Please include this amended Role of Funder statement in your cover letter; we will change the online submission form on your behalf.

**Reply:** The funders had no role in the research. As stated in the cover letter: "The funders had no role in study design, data collection and analysis, decision to publish, or preparation of the manuscript."

**Requirement 3:** Please remove all personal information, ensure that the data shared are in accordance with participant consent, and re-upload a fully anonymized data set.

Note: spreadsheet columns with personal information must be removed and not hidden as all hidden columns will appear in the published file.

**Reply:** All uploaded data were removed personal information and obtained participant consent. These protocols were stated in the "Ethics Statement" subsection of the original manuscript, as follows: "Written informed consent was obtained from individual participants or their guardians. Records were accessed on 20th March 2022 and subsequently anonymized to protect the personal privacy of all pregnant women.".

**Requirement 4:** Please include captions for your Supporting Information files at the end of your manuscript, and update any in-text citations to match accordingly.

**Reply:** The captions for the Supporting Information files have been added in the "Supporting Information" section at the end of the manuscript, and the in-text citations have been updated accordingly.

**Reviewers' comments:**

**Reviewer #1**

**Comment 5:** First, the manuscript does not report the test failure rate or the number of samples for which results were inconclusive or uninterpretable. This information is essential to assess the practical applicability and reliability of the test in a real-world clinical setting.

**Reply:** We retrospectively reviewed genome-wide non-invasive prenatal testing (GW-NIPT) results from November 2017 to May 2021, and found that a total of 106 cases failed due to fetal fraction below 3.5%. Thus, a total of 59,877 patients underwent testing, with 59,771 patients ultimately included in the analysis. In the manuscript, we made corresponding modifications in the "Subjects and Methods", "Results" sections and Figure 1.

(1)Subjects and Methods Section

In the "Study subjects" subsection, We change the first sentence into: "The retrospective study enrolled 59,877 singleton or twin pregnant women opting for GW-NIPT as a first-tier prenatal test from November 2017 to May 2021 at Shenzhen Baoan Women's and Children's Hospital in China. Among these, 59,771 women were included in the subsequent analysis due to 106 test failures."

(2) Results Section

We revised the heading of the first section to "Study population characteristics and GW-NIPT results", and added the following sentence described the test failure rate: "A total of 59,877 pregnancies who underwent GW-NIPT from November 2017 to May 2021 were accessioned for this study and 106 were tested failure (fetal fraction <3.5%). The test failure rate was 0.18% (106/59877) and 59,771 cases were enrolled in further analysis (Fig 1)."

(3) Figure 1

**Fig 1. Flowchart of the GW-NIPT results and outcomes of pregnant women.** GW-NIPT, genome-wide non-invasive prenatal test; n, number; T, trisomy; SCA, sex chromosome aneuploidy; RAA, rare autosomal aneuploidy; CNV, copy number variation; CMA, chromosomal microarray analysis; FISH, fluorescence in situ hybridization. ^*^Include 1,315 cases that underwent both karyotyping and CMA.

**Comment 6:** Second, while the authors report performance metrics such as sensitivity and specificity, the methodology behind these calculations is not sufficiently explained. It is unclear how true positives were defined and whether confirmation was based on invasive diagnostic testing, postnatal outcomes, or clinical evaluation. A detailed description of the reference standard and classification criteria is necessary to validate the reported performance.

**Reply:** In terms of the definitions of true positives, false positives, etc., associated with the calculation of performance metrics such as sensitivity and specificity, we have modified Figure 1 (See Comment 5) to make the NIPT results and follow-up process clearer and more visible, and offered a detailed explanation in the "Subjects and Methods" section.

In the subsection of "Invasive prenatal diagnosis and postnatal outcomes", we modified the first paragraph to "Pregnant women who required further examination would have amniocentesis for karyotyping, chromosomal microarray analysis (CMA), or fluorescence in situ hybridization (FISH), following the results of NIPT, ultrasound examination and other medical history. A small number of cases underwent diagnostic testing using chorionic villus sampling (CVS), umbilical cord blood, and fetal tissue." We aimed to explicitly state that invasive prenatal diagnosis included karyotyping, CMA, and FISH. These diagnostic tests can be selected individually or in combination by doctors and patients based on comprehensive evaluation.

We also added two following paragraphs in this section to describe the postnatal outcomes and classification criteria for the results:

"For postnatal outcomes, we included information on the types of delivery (live birth vs. stillbirth), the presence of any birth defects, and the clinical evaluation of birth defects. When postnatal outcomes was not recorded, potential reasons included termination of pregnancy (TOP) or delivery at another hospital, which we did not differentiate in this study."

"We classified NIPT results into true positive (TP), false positive (FP), true negative (TN) and false negative (FN) according to invasive testing results or the postnatal outcomes. The criteria for judgment of NIPT results are as follows: (1) T21, T18, T13 and RAA can be comfirmed via invasive testing (karyotyping, CMA or FISH) or postnatal outcomes due to their distinct clinical features, such as atelencephalia in common trisomies and cardiovascular defects in RAA [22, 23]. (2) SCA was comfirmed by invasive testing of karyotyping, CMA or FISH. (3) CNV (≥5 Mb) was comfirmed by invasive testing of CMA."

References:

22. Syngelaki A, Guerra L, Ceccacci I, Efeturk T, Nicolaides KH. Impact of holoprosencephaly, exomphalos, megacystis and increased nuchal translucency on first-trimester screening for chromosomal abnormalities. Ultrasound Obstet Gynecol. 2017;50(1):45-48.

23. Hu R, Huang W, Zhou W, et al. Phenotypic findings and pregnancy outcomes of fetal rare autosomal aneuploidies detected using chromosomal microarray analysis. Hum Genomics. 2022;16(1):64.

**Comment 7:** Additionally, although the study employs a genome-wide approach, the discussion could better emphasize the advantages of this strategy. Specifically, the authors should elaborate on how genome-wide NIPT may increase diagnostic yield by detecting atypical, rare, or subchromosomal CNVs that would not be identified by targeted assays. This is a key strength of the approach and should be clearly articulated.

**Reply:** We added a comparative analysis between GW-NIPT and targeted CMA in the final paragraph of the Discussion section:

"Based on targeted array CGH using Fetal DNA Chip Version 1.2, CMA can detect all common aneuploidy diseases and over 100 types of MMSs of known gene sequence, yet has limitations like missing some small chromosomal abnormalities, copy number polymorphisms, mosaicism, and rearrangements. GW-NIPT enables detection of a broader range of genomic variations and is capable of identifying atypical, rare, or subchromosomal CNVs that would not be identified by targeted assays. The Algorithms of GW-NIPT developed by BGI-Shenzhen were used to detect CNV and the reads were aligned to the human genome (hg19). After raw data processing, GC correction, intra-batch correction and Principal Component Analysis (PCA), Hidden Markov Model (HMM) was used to infer the hidden states of each window (normal, deletion and duplication) [40]. Concurrently, GW-NIPT identified 2 cases of SCA mosaicism (comfirmed by karyotyping) that were undetected by CMA. These findings underscore the clinical necessity of implementing GW-NIPT."

References:

40. Chen S, Zhang L, Gao J, et al. Expanding the Scope of Non-invasive Prenatal Testing to Detect Fetal Chromosomal Copy Number Variations. Front Mol Biosci. 2021;8:649169.

**Comment 8:** Importantly, the manuscript does not specify the resolution limit for CNV detection (i.e., the minimum size of CNVs that can be reliably identified). This technical parameter is critical for interpreting the scope and limitations of the assay and for comparing its performance with other technologies such as array-CGH. The authors should clearly indicate this resolution threshold and how it was validated.

**Reply:** This retrospective study evaluated the performance of GW-NIPT in a clinical setting. To minimize unnecessary invasive procedures, only CNVs ≥5 Mb were reported in the test results. For a more direct expression, we have changed it to "CNV (≥5 Mb)" in the entire manuscript.

There are several relevant articles supporting this resolution limit for CNV detection. R Li, et al. reported that among CMA-positive samples, the detection rate of NIPT was 90.9% for CNVs >5 Mb, compared to only 14.3% for CNVs <5 Mb. [41]. Furthermore, studies have reported that for CNVs <5 Mb detected by expanded NIPT, the positive predictive values (PPVs) were only approximately 20%, whereas for CNVs ≥5 Mb, the PPVs ranged from 30% to 70% [14, 19].

Additionally, although CMA testing in invasive prenatal diagnosis may report clinically significant microduplications or microdeletions greater than 100kb of genomic copy number, we restricted our comparison between NIPT and CMA to cases where NIPT was negative for CNVs but CMA was positive—including only those with CMA-detected CNVs ≥5 Mb.

References:

14. Xue H, Yu A, Lin M, et al. Efficiency of expanded noninvasive prenatal testing in the detection of fetal subchromosomal microdeletion and microduplication in a cohort of 31,256 single pregnancies. Sci Rep. 2022;12(1):19750.

19. Li C, Xiong M, Zhan Y, et al. Clinical Potential of Expanded Noninvasive Prenatal Testing for Detection of Aneuploidies and Microdeletion/Microduplication Syndromes. Mol Diagn Ther. 2023;27(6):769-779.

41. Li R, Wan J, Zhang Y, et al. Detection of fetal copy number variants by non-invasive prenatal testing for common aneuploidies. Ultrasound Obstet Gynecol. 2016;47(1):53-57.

**Comment 9:** Furthermore, a demographic summary of the study cohort—including maternal age, gestational age, and clinical indications for testing—would provide valuable context for the reader. The authors are also encouraged to include more detail on the bioinformatic workflow used for CNV calling, including the software tools, filtering criteria, and any validation methods applied. Sharing access to the analysis pipeline, when possible, would greatly enhance the study's transparency and reproducibility.

**Reply:** A demographic summary of the study cohort—including maternal age, body mass index (BMI), gestational age, and pregnancy types, was presented in Table 1. We have made the following modifications in the "Results" section:

"Demographic characteristics of the study population were summarized in Table 1. The average maternal age was 29.5 (±4.1) years which is slightly higher than the national average age of pregnant women of 28.8 (±5.1) years from 2016 to 2020 [24], and 11.6% (6946/59771) of pregnant women were in the advanced maternal age (≥35). The mean body mass index (BMI) was 21.7 (±3.0) kg/m2 and the median gestational age was 17 weeks (range: 12-39.3)."

Additionally, the bioinformatics software used for analyzing CNVs and other chromosomal abnormalities was BGI HALOS, as mentioned in the “Laboratory analysis of GW-NIPT” of the “Subjects and Methods” section. We added following description of the detailed bioinformatics workflow used for CNV calling in the last paragraph of the "Discussion" section:

"GW-NIPT enables detection of a broader range of genomic variations and is capable of identifying atypical, rare, or subchromosomal CNVs that would not be identified by targeted assays. The Algorithms of GW-NIPT developed by BGI-Shenzhen were used to detect CNV and the reads were aligned to the human genome (hg19). After raw data processing, GC correction, intra-batch correction and Principal Component Analysis (PCA), Hidden Markov Model (HMM) was used to infer the hidden states of each window (normal, deletion and duplication) [40]."

**Table 1. Demographic characteristics of the the study cohort.** n, number; BMI, body mass index.

| **Maternal characteristics** | **n (%)** |
| --- | --- |
| *Maternal age (years)* |  |
| < 35 | 52825 (88.4) |
| ≥ 35 | 6946 (11.6) |
| *BMI (kg/m2)* |  |
| < 18.5 | 6469 (10.8) |
| 18.5 - 23.9 | 41179 (68.9) |
| 24.0 - 27.9 | 10001 (16.7) |
| ≥ 28.0 | 2109 (3.5) |
| Unrecorded | 13 (0.0) |
| *Gestation age (weeks)* |  |
| < 28 | 58928 (98.6) |
| ≥ 28 | 835 (1.4) |
| Unrecorded | 8 (0.0) |
| *Pregnancy types* |  |
| Singleton pregnancy | 58342 (97.6) |
| Twin pregnancy | 1421 (2.4) |
| Unrecorded | 8 (0.0) |

References:

24. Zhou Y, Yu H, Wang A, et al. Temporal and geographic trends in maternal and paternal ages in China from 2016 to 2020. Chinese Journal of Reproductive Health. 2024;35(4):301-305, 312.

40. Chen S, Zhang L, Gao J, et al. Expanding the Scope of Non-invasive Prenatal Testing to Detect Fetal Chromosomal Copy Number Variations. Front Mol Biosci. 2021;8:649169.

**Comment 10:** Finally, some minor editorial improvements could enhance the clarity and flow of the text, particularly in the abstract and results sections.

**Reply:** We have made several revisions to enhance the clarity and flow of the text, particularly in the abstract and results sections.

(1) Abstract Section

We replaced "NIPT" with "GW-NIPT" to explicitly denote genome-wide non-invasive prenatal testing. In the subsection of results, Our modifications are as follows: "Among 59,877 pregnant women who underwent GW-NIPT, 59,771 were successfully tested. Of these, 499 (0.83%) were identified with 504 high-risk fetal chromosomal abnormalities, including 5 cases each carrying two distinct abnormalities. Follow-up analysis demonstrated that GW-NIPT sensitivity exceeded 97% for fetal aneuploidies and was 63.6% for CNV (≥5 Mb). The positive predictive values for T21, T18, T13, sex chromosome aneuploidy, rare autosomal aneuploidy, and CNV (≥5 Mb) were calculated as 83.1%, 25.8%, 10.3%, 51.9%, 2.0%, and 33.9%, respectively."

(2) Results Section

The "Study population characteristics and GW-NIPT results" subsection presents the illustration of the overall NIPT results and diagnostic process, as follows:

"A total of 59,877 pregnancies who underwent GW-NIPT from November 2017 to May 2021 were accessioned for this study and 106 were tested failure (fetal fraction <3.5%). The test failure rate was 0.18% (106/59877) and 59,771 cases were enrolled in further analysis (Fig 1). In the results of GW-NIPT, 499 (0.8%) patients were suspected to be high risk, of which 5 carried two types of abnormal high risk. Among 504 high-risk cases, 367 cases were confirmed by follow-up diagnosis, and 137 cases were lost to follow-up. 59,272 cases showed low-risk results for all of the detected chromosomal abnormalities, of which 1,402 cases underwent invasive CMA and 1,349 cases conducted invasive karyotype analysis based on other clinical evaluations, including 1,315 cases that underwent both CMA and karyotyping. Among the remaining low-risk cases of NIPT, 43,413 have recorded postnatal outcomes and 14,423 cases lost to follow-up."

We incorporated the analysis of GW-NIPT performance for all test results into Table 2, and added low-risk data and specificity of NIPT for each type of anomaly. Corresponding minor modifications were made in the subsections of "Performance of GW-NIPT for screening common trisomies" and "Performance of GW-NIPT for screening SCAs, RAAs and CNVs (≥5 Mb)", as seen in the original manuscript, which will not be listed here.

(3) Discussion Section

The fourth paragraph has been slightly edited for improved clarity and conciseness: "Kim H et al. reported comparable NIPT PPV for overall SCA (51.4%), with subtype-specific values of 88.9% (47,XXX), 71.4% (47,XXY), 60.0% (47,XYY), and 18.8% (45,X) [11]. Our results demonstrated similar trends: 58.8% (47,XXX), 90.9% (47,XXY), 83.3% (47,XYY), and 19.7% (45,X)."

References:

11. Kim H, Park JE, Kang KM, et al. Clinical evaluation of noninvasive prenatal testing for sex chromosome aneuploidies in 9,176 Korean pregnant women: a single-center retrospective study. BMC Pregnancy Childbirth. 2024;24(1):93.

**Table 2. NIPT performance for common trisomies, SCA, RAA and CNV.** NIPT, non-invasive prenatal test; n, number; TP, true positive; FP, false positive; FN, false negative; CI, confidence interval; PPV, positive predictive value; T, trisomy. SCA, sex chromosome aneuploidy; RAA, rare autosomal aneuploid; CNV, copy number variation. ^*^Confirmed as trisomy 21 mosaicism. ^$^Confirmed as SCA mosaicisms.

| **Abnormal types** | **NIPT high risk** | | | | **NIPT low risk** | | | | **Sensitivity, %**  **(95% CI)** | **Specificity, %**  **(95% CI)** | **PPV, %**  **(95% CI)** |
| --- | --- | --- | --- | --- | --- | --- | --- | --- | --- | --- | --- |
|  | **n** | **lost** | **TP** | **FP** | **n** | **lost** | **FN** | **TN** |  |  |  |
| T21 | 89 | 24 | 54 | 11 | 59684 | 14475 | 1^*^ | 45208 | 98.2 (94.6-100) | 100 (99.9-100) | 83.1 (74.0-92.2) |
| T18 | 36 | 5 | 8 | 23 | 59735 | 14494 | 0 | 45243 | 100 (67.6-100) | 99.9 (99.9-100) | 25.8 (10.4-41.2) |
| T13 | 33 | 4 | 3 | 26 | 59738 | 14495 | 0 | 45245 | 100 (43.8-100) | 99.9 (99.9-100) | 10.3 (0-21.4) |
| SCA | 194 | 65 | 67 | 62 | 59577 | 57942 | 2^$^ | 1633 | 97.1 (93.1-100) | 96.3 (95.4-97.2) | 51.9 (43.3-60.6) |
| RAA | 61 | 10 | 1 | 50 | 59710 | 14489 | 0 | 45223 | 100 (20.6-100) | 99.9 (99.8-99.9) | 2.0 (0-5.8) |
| CNV | 91 | 29 | 21 | 41 | 59680 | 58032 | 12 | 1636 | 63.6 (47.2-80.0) | 97.6 (96.8-98.3) | 33.9 (22.1-45.6) |

**Reviewer #2**

**Comment 11:** The study of 59,771 pregnant women in the general obstetric population substantiated a low specificity for fetal trisomy 21, 18, and 13 with PPV of 83.1%, 25.8%, and 10.3%, respectively.

On the contrary the technical validation of the method (DOI: 10.1002/uog.16010) reported a high specificity with PPV of 99.98, 99.98, 99.9 for T21, T18, T13 respectively.

We note that the analysis of the study performance shows good sensitivity while the specificity is particularly low both with respect to the technical validation of the method and to international data.

This important difference raises a question of reliability that requires an answer. The authors compare the difference in specificity with an insufficient number of published case studies and suggest uncertain motivations. For example, the recent case study on approximately 72,000 consecutive cases from a single center in two years (DOI: 10.1002/pd.6580) performed with a different technique (no PCR, pair end sequencing) that reports PPV 99.3, 98.9, 82.22 for T21, T18, T13 respectively, was not considered.

Since low specificity is one of the limiting parameters in the cost-benefit evaluation of NIPT use leading to the execution of an improper number of invasive controls, the authors are asked for a complete review of the case studies and a reassessment of the conclusions regarding the reliability of the method used in the study.

**Reply:** This article is a retrospective study of clinical data generated through standard clinical procedures, with all results reported to patients according to routine practice. However, our analysis revealed a relatively significant number of false positives for high-risk T21, T18, and T13 of NIPT. These false-positive cases have been comprehensively documented in S1 Table. Therefore, our calculated PPVs for GW-NIPT in detecting T21, T18, and T13 were 83.08%, 25.81%, and 10.34% respectively, which are lower than the PPVs reported in these two articles (DOI:10.1002/uog.16010 and DOI: 10.1002/pd.6580). Differences between our research and the two aforementioned articles are listed in Table A, including the sensitivity, specificity and PPV of NIPT, as well as study population, sample size, sample source, testing principles, NIPT Platform, data processing software and sequencing depth. These variations likely represent key contributors to the observed discrepancies in PPV values.

Specifically, in this article and the report by Ma, J et al. (DOI: 10.1002/uog.16010), although the detection principles are similar, the platforms are different. Compared to the BGISEQ-1000 platform, the BGISEQ-500 has relatively lower operating costs, with the cost per gigabase (Gb) approximately 40% -60% of that of the Illumina HiSeq 4000 platform [42]. In addition to common trisomies, the BGISEQ-500 platform demonstrates expanded detection capabilities for SCA, RAA, CNV, etc., supporting diverse clinical applications. Moreover, direct comparisons across studies remain challenging due to different study objectives and methodological variations in data processing pipelines and cohort characteristics. In addition, Lu YS et al. reported that the PPVs of T21, T18 and T13 detected by NIPT (a cohort of 263,744 pregnancies) using BGI-500/2000 were 84.1%, 43.6%, and 16.4%, respectively [43], which are similar to the results of our study.

We also recognized certain limitations in this study, as noted in the Discussion secton: "This study has several limitations: (1) Incomplete postnatal outcomes, without distinguishing between TOP and lost to follow-up cases. (2) Long-term follow-up was not performed, and no data were collected on the long-term impact of NIPT results on neonatal outcomes or childhood health. (3) In-depth analysis on high-risk pregnancies—particularly those with maternal morbidity or negative neonatal outcomes—was insufficiently conducted. These methodological gaps should be prioritized in subsequent research to enhance the robustness of future studies."

**Table A. Comparison between different studies.** T, trisomy; PPV, positive predictive value.

|  | **Our research** | **Ma, J et al.**  **DOI: 10.1002/uog.16010** | | **Faieta M, et al.**  **DOI: 10.1002/pd.6580** |
| --- | --- | --- | --- | --- |
|  |  | retrospective group | prospective group |  |
| **Sensitivity (%)** |  |  |  |  |
| T21 | 98.2 | 100 | 100 | 99.5 |
| T18 | 100 | 100 | 100 | 100 |
| T13 | 100 | 100 | 100 | 100 |
| **Specificity (%)** |  |  |  |  |
| T21 | 100 | 100 | 100 | 100 |
| T18 | 99.9 | 100 | 100 | 100 |
| T13 | 99.9 | 100 | 100 | 100 |
| **PPV (%)** |  |  |  |  |
| T21 | 83.1 | 98.4 | 100 | 99.3 |
| T18 | 25.8 | 95 | 100 | 98.9 |
| T13 | 10.3 | 100 | 100 | 82.2 |
| **Study population** | General obstetrics pregnant women | 8155 pregnancies | 2439 high-risk  pregnancies | General obstetrics pregnant women |
| **Sample size** | 59771 | 10594 | | 71883 |
| **Sample source** | A single center | 20 centers | | A single center |
| **Testing Principles** | combinatorial probe-anchor synthesis | combinatorial probe-anchor ligation | | paired‐end whole‐genome sequencing assay |
| **NIPT Platform** | BGISEQ-500 | BGISEQ-1000 | | Veriseq NIPT Solution v2 (Illumina) |
| **Data processing software** | HALOS-NIFTY_CN-V4.1.5 | - | | Veriseq v2 |
| **Sequencing depth** | 0.17 × | - | | - |

References:

42. Natarajan KN, Miao Z, Jiang M, et al. Comparative analysis of sequencing technologies for single-cell transcriptomics. Genome Biol. 2019;20(1):70.

43. Lu YS, Chen YY, Ding SY, et al. Performance analysis of non-invasive prenatal testing for trisomy 13, 18, and 21: A large-scale retrospective study (2018-2021). Heliyon. 2024;10(13):e33437.

**Reviewer #3**

**Comment 12:** It is not entirely clear whether follow-up results were available for all cases identified as high- or low-risk by NIPT. Clarifying the extent and completeness of follow-up data.

**Reply:** Follow-up comprised both invasive testing (karyotyping, CMA and FISH) and postnatal outcomes. Among the enrolled cases, both partial and complete loss to follow-up cases were observed, with the detailed tracking flowchart presented in Figure 1 (See Comment 5).

Table 2 (See Comment 10) further stratifies the follow-up results by specific chromosomal abnormalities. Negative cases may include high-risk findings of other chromosomal abnormalities, thereby allowing for a direct illustration of the calculation process for sensitivity, specificity, and PPV.

**Comment 13:** In some sections, the technical language is unclear. For example, in the cytogenetic analysis, 'karyotypes' are mentioned instead of 'metaphase' (in the paragraph titled Invasive prenatal diagnosis).

**Reply:** After careful review, we have made the following modifications to the entire text.

(1) Laboratory analysis of NIPT Section

The concentration range of DNA nanoball (DNB) was systematically adjusted as follows: "DNB concentrations in the range of 8-40 ng/μl was considered qualified."

(2) Invasive prenatal diagnosis and postnatal outcomes Subsection

We employed cells arrested at metaphase, with the following experimental modifications: "After standard metaphase conversion of cultured fetal cells, chromosome analysis was performed on at least 20 metaphase cells of each specimen, while each mosaic specimen was counted in more than 30 metaphase cells." Meanwhile, we have provided additional clarification on CMA as follows: "CMA was conducted using comparative genomic hybridization (CGH) technology."

(3) Statistical analysis Subsection

We have comprehensively detailed the statistical approaches, with the following revisions: "Sensitivity, specificity and PPV were calculated using 2×2 tables with following formula: sensitivity=TP/(TP+FN), specificity=TN/(TN+FP) and PPV=TP/(TP+FP). On the bases of a standard normal distribution, 95% confidence intervals (CI) were calculated by Normal Approximation method and Wilson method."

**Comment 14:** In the classification of results, 'No Calls' are not mentioned.

**Reply:** In this article, additional data were further supplemented to improve the authenticity of clinical test information. Upon retrospective review, a total of 59,877 pregnant women applied for NIPT between November 2017 and May 2021. Among these, 106 cases (0.18%) resulted in test failure (No Calls) due to fetal fraction below 3.5%. In the manuscript, we made corresponding modifications in the "Subjects and Methods", "Results" sections and Figure 1 (See Comment 5).

(1)Subjects and Methods Section

In the "Study subjects" subsection, We change the first sentence into: "The retrospective study enrolled 59,877 singleton or twin pregnant women opting for GW-NIPT as a first-tier prenatal test from November 2017 to May 2021 at Shenzhen Baoan Women's and Children's Hospital in China. Among these, 59,771 women were included in the subsequent analysis due to 106 test failures."

(2) Results Section

We revised the heading of the first section to "Study population characteristics and GW-NIPT results", and add a sentence describing the test failure rate: "A total of 59,877 pregnancies who underwent GW-NIPT from November 2017 to May 2021 were accessioned for this study and 106 were tested failure (fetal fraction <3.5%). The test failure rate was 0.18% (106/59877) and 59,771 cases were enrolled in further analysis (Fig 1)."

**Comment 15:** There is a lack of classification for the detected CNVs, distinguishing between pathogenic and non-pathogenic.

**Reply:** Regarding abnormal CNVs from follow-up diagnostics, we classified variants into two categories: "pathogenic/likely pathogenic (P/LP)" and "variant of uncertain significance (VUS)", according to ACMG's recommendation in 2015 [21]. Specifically, we evaluated the pathogenicity of these mutated genes through the Population, Disease-Specific, and Sequence Databases, provided relevant cases and explanations of potential diseases based on published literature, and advised doctors to conduct a comprehensive evaluation of patients.

(1) Subjects and Methods Section

In the Subsection "Invasive Prenatal Diagnosis and Postnatal Outcomes", the following paragraph can be added to explain the classification for the detected CNVs: "Based on the evaluation of the pathogenicity of genetic variants referring to Population, Disease-Specific, and Sequence Databases, as well as published literature, CNVs were categorized into two groups: "pathogenic/likely pathogenic (P/LP)" and "variant of uncertain significance (VUS)".

(2) Results Section

The following sentence was added to the last paragraph of "Performance of GW-NIPT for screening SCAs, RAAs, and CNVs (≥5 Mb)" to describe the outcomes of P/LP and VUS CNVs: "Regarding comfirmed CNV, more adverse postnatal outcomes occurred in cases with P/LP variants compared to cases with VUS variants, including premature birth, stillbirth or possible TOP (S2 and S5 Table)." Meanwhile, a column named "Classification of CNVs" was added in S2 and S5 Table to supplement the pathogenicity classification of CMA results.

(3) Discussion Section

Further analysis was conducted on the detection of CNVs with different pathogenicities and the potential reasons for the differences in PPVs. We recommend that live-born fetuses with high-risk CNVs should undergo long-term follow-up after birth. Revise in the seventh paragraph as follows:

"Additionally, among 91 cases with high-risk CNV (≥5 Mb) indications by NIPT, we identified 17 cases of MMSs. Among these cases, three were confirmed as true positives, including one case each of Wolf-Hirschhorn syndrome, 18q deletion syndrome, and Cri-du-Chat syndrome (CDC). This indicates that GW-NIPT is capable of detecting pathogenic MMS, albeit with a low true-positive rate. Studies report variable PPVs of NIPT for specific MMS.For example, Xue H et al. found PPVs of 50%, 50% and 0% for CDC, PWS/AS and 1p36 deletion, respectively [14] and Petersen et al. reported corresponding PPVs of 0%, 0% and 14% [36].These may be influenced by factors such as CNVs carried by pregnant woman, sequencing coverage, bioinformatics algorithms, fetal DNA fraction, etc. [37]. Furthermore, our results demonstrate that fetuses with P/LP CNVs were more likely to result in TOP or adverse postnatal outcomes compared to those with VUS CNVs. Besides, long-term follow-up and monitoring should be implemented for cases with high-risk CNVs who continue to live birth."

References:

14. Xue H, Yu A, Lin M, et al. Efficiency of expanded noninvasive prenatal testing in the detection of fetal subchromosomal microdeletion and microduplication in a cohort of 31,256 single pregnancies. Sci Rep. 2022;12(1):19750.

21. Richards S, Aziz N, Bale S, et al. Standards and guidelines for the interpretation of sequence variants: a joint consensus recommendation of the American College of Medical Genetics and Genomics and the Association for Molecular Pathology. Genet Med. 2015;17(5):405-424.

36. Petersen AK, Cheung SW, Smith JL, et al. Positive predictive value estimates for cell-free noninvasive prenatal screening from data of a large referral genetic diagnostic laboratory. Am J Obstet Gynecol. 2017;217(6):691.e1-691.e6.

37. Duan H, Wang W, Zhang Y, Chen X, Jiang Z, Li J. Accuracy of expanded noninvasive prenatal testing for maternal copy number variations: A comparative study with CNV-seq of maternal lymphocyte DNA. Taiwan J Obstet Gynecol. 2024;63(4):536-539.

**Comment 16:** It is unclear how the false negative rate was calculated, given the absence of follow-up data.

**Reply:** Through further rigorous data analysis, we have detailed follow-up data in Figure 1, Table 2, and the corresponding original manuscript.

(1) Figure 1 (See Comment 5)

Based on NIPT results, we stratified patients with successful testing into high-risk (indicating potential abnormalities) and low-risk (no disease indication) groups, with their respective follow-up outcomes documented. The original manuscript described this in "Study population characteristics and GW-NIPT results" subsection as follows:

"A total of 59,877 pregnancies who underwent GW-NIPT from November 2017 to May 2021 were accessioned for this study and 106 were tested failure (fetal fraction <3.5%). The test failure rate was 0.18% (106/59877) and 59,771 cases were enrolled in further analysis (Fig 1). In the results of GW-NIPT, 499 (0.8%) patients were suspected to be high risk, of which 5 carried two types of abnormal high risk. Among 504 high-risk cases, 367 cases were confirmed by follow-up diagnosis, and 137 cases were lost to follow-up. 59,272 cases showed low-risk results for all of the detected chromosomal abnormalities, of which 1,402 cases underwent invasive CMA and 1,349 cases conducted invasive karyotype analysis based on other clinical evaluations, including 1,315 cases that underwent both CMA and karyotyping. Among the remaining low-risk cases of NIPT, 43,413 have recorded postnatal outcomes and 14,423 cases lost to follow-up."

(2) Table 2 (See Comment 10)

For each chromosomal abnormality type, calculations were performed independently by subtracting high-risk case numbers from the total cohort to derive corresponding low-risk case numbers. Using classification criteria for different abnormality types (refer to Comment 6), true-negative and false-negative cases were determined, from which specificity was calculated. These data are comprehensively presented in Table 2. Therefore, several minor modifications were made in subsections "Performance of GW-NIPT for screening common trisomies" and "Performance of GW-NIPT for screening SCAs, RAAs and CNVs (≥5 Mb)", as seen in the original manuscript, which will not be listed here.

**Comment 17:** Additionally, there are several considerations that could be addressed to strengthen the findings:

Long-Term Follow-Up: Although some follow-up results have been described, the study does not seem to include long-term data on how the NIPT results have actually impacted neonatal outcomes or the health of children in the long term.

Lack of In-Depth Analysis on High-Risk Pregnancies: Data on outcomes for high-risk pregnancies, such as those with maternal morbidity or negative neonatal outcomes, are poorly addressed. These data could enhance the understanding of the usefulness of NIPT not only in normal pregnancies but also in high-risk situations.

**Reply:** These represent a limitation of our current study. We have added a discussion of this shortcoming in the final paragraph of the Discussion section: "This study has several limitations: (1) Incomplete postnatal outcomes, without distinguishing between TOP and lost to follow-up cases. (2) Long-term follow-up was not performed, and no data were collected on the long-term impact of NIPT results on neonatal outcomes or childhood health. (3) In-depth analysis on high-risk pregnancies—particularly those with maternal morbidity or negative neonatal outcomes—was insufficiently conducted. These methodological gaps should be prioritized in subsequent research to enhance the robustness of future studies."

We have addressed all the journal requirements and reviewers' comments as outlined above. The revisions have been carefully incorporated into the manuscript. Should there be any further issues or additional revisions required, please do not hesitate to contact us.

Yours sincerely,

Jianxin Zhen

Central Laboratory

Shenzhen Baoan Women's and Children's Hospital

Shenzhen, Guangdong 518102

China

Tel: +86-075527863999

E-mail: jxzhen@qq.com
